# The first complete mitochondrial genome sequence of the korean endemic catfish *Silurus microdorsalis* (Actinopteri, Siluriformes, Siluridae)

**DOI:** 10.1080/23802359.2019.1698336

**Published:** 2019-12-13

**Authors:** Chang Eon Park, Yeong-Jun Park, Min-Chul Kim, Min-Kyu Park, YeonGyun Jung, Seung-Dae Choi, YoungJae Jo, Gi-Ung Kang, Min-Ji Kim, Qing X. Li, Brandon A. Yoza, Kgu-Hwan Kim, Hee Cheon Park, Jae-Ho Shin

**Affiliations:** a School of Applied Biosciences, Kyungpook National University, Daegu, Republic of Korea;; b Institute of Ornithology, Association of Ex-situ Conservation, Daegu, Republic of Korea;; c Department of Molecular Biosciences and Bioengineering, University of Hawaii at Manoa, Honolulu, HI, USA;; d Hawaii Natural Energy Institute, University of Hawaii at Manoa, Honolulu, HI, USA;; e Department of Radiologic Technology, Daegu Health College, Daegu, Republic of Korea

**Keywords:** *Silurus microdorsalis*, Korean endemic catfish, Siluridae

## Abstract

The *Silurus microdorsalis* is known as Korean endemic slender catfish. Despite its value as a biological resource, there is no complete mitochondrial genome sequence. The complete mitochondrial genome consisted of 16,524 bp including 22 transfer RNA (tRNAs), 2 ribosomal RNA (rRNAs), 13 protein-coding genes (PCGs), and A + T rich region. The overall base composition of *S. microdorsalis* was A + T: 56.1%, C + G: 43.9%, apparently with slight AT bias. Phylogenetic relationship showed that *S. microdorsalis* was closely related to *Silurus glanis.*

The Korean endemic catfish *Silurus microdorsalis* (Actinopteri, Siluriformes, Siluridae) is classified in Siluridae family and the only inhabits in Korea (Jeon 1984). Mostly, the catfish was inhabited in the muddy floor of the river. However, *S. microdorsalis* is found in only mountainous streams (Park and Kim 2005). Furthermore, there is a rise in the value of *S. microdorsalis* because of its possibility as fresh water aquaculture species (Ki and Lee 2018). Even if it has a value as biological resource, genetic study of *S. microdorsalis* is not reported. In this study, we sequenced mitochondrial genome (mitogenome) and analyze phylogenetic relationship with other catfish.

A sample of *S. microdorsalis* was collected from Wangpicheon river, Kyungsangpook-do, Republic of Korea (GIS: 36°57′10.4″N 129°22′03.5″E). This specimen was deposited at the Institute of Ornithology, Kyungpook National University, Daegu, Republic of Korea. Also, the whole-body specimen is being kept under the voucher number NIBRP0000006493 at National Institute of Biological Resources, Republic of Korea. The mitogenome was amplified into two long amplicon by long-PCR method (Koh et al. 2018). DNA shotgun sequencing was proceeded according to the manual of Ion Torrent PGM platform (Thermo Fisher Scientific, Waltham, MA, USA). Then, mitogenome was assembled and annotated using the CLC genomics workbench version 7.5 (CLC Bio, Denmark).

The mitogenome was composed of circular DNA with 16,524 bp containing 22 transfer RNA (tRNAs), 2 ribosomal RNA (rRNAs), 13 protein-coding gene (PCGs), and A + T rich region. In PCGs, the start codon ATG is mainly used for PCGs transcription. Only, *cox1* use GTG for start codon. In case of stop codon, TAA is used for stop codon except *nd2*, *cox2*, *cox3*, *nd3*, *nd4*, and *cytb* which terminated by incomplete stop codon T(aa) (Zhang et al. 2016). The nucleotide composition of assembled mitogenome is asymmetric (A: 30.3%, T: 25.8%, G: 15.8%, C: 28.1%) with a slight AT bias. The complete mitogenome of *S. microdorsalis* was submitted to GenBank (accession number: NC028175).

The phylogenetic relationship of *S. microdorsalis* was compared with previously researched mitogenome on *Silurus*, *Kryptopterus*, *Ompok*, and *Pterocryptis* genus in Siluridae family (Vittas et al. 2011; Xu et al. 2016; Barman et al. 2017). The phylogenetic tree was illustrated using maximum likelihood general time reversible model (GTR) with gamma-distributed (G) plus invariant sites (I) method with 1000 replicate bootstrap (Park et al. 2019). The phylogenetic location of *S. microdorsalis* was close to *Silurus glanis* which inhabited the Europe area (Figure 1). Morphological and anatomical characters are used in phylogenetic classification of catfish (Ünlü et al. 2012). However, according to this result, mitogenome information can be used as one of the tools for phylogenetic classification of catfish. This result can contribute to phylogenetic relationship of the genus *Silurus*.

**Figure 1.**
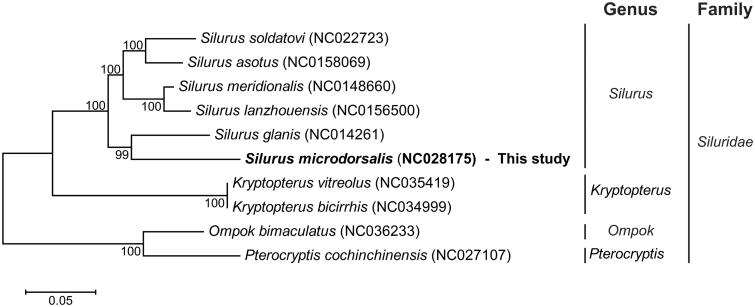
Phylogenetic tree of *Silurus microdorsalis* with other catfish in Siluridae family. The bootstrap values were written on the phylogenetic tree. On the right side, vertical stick indicated genus and family information of catfish previously reported. The GenBank accession numbers are mentioned next to the nomenclature.
